# Robust Prediction of Prognosis and Immunotherapy Response for Bladder Cancer through Machine Learning Algorithm

**DOI:** 10.3390/genes13061073

**Published:** 2022-06-16

**Authors:** Shanshan Hu, Shengying Gu, Shuowen Wang, Chendong Qi, Chenyang Shi, Fengdan Qian, Guorong Fan

**Affiliations:** Department of Clinical Pharmacy, Shanghai General Hospital, Shanghai Jiao Tong University School of Medicine, Shanghai 200080, China; h1435086126@163.com (S.H.); gsy15815@163.com (S.G.); wangshuowensy@163.com (S.W.); poohqi25@163.com (C.Q.); shicheny@163.com (C.S.); qianfengdan@126.com (F.Q.)

**Keywords:** bladder cancer, machine learning, immunotherapy, prognosis, ferroptosis

## Abstract

The important roles of machine learning and ferroptosis in bladder cancer (BCa) are still poorly understood. In this study, a comprehensive analysis of 19 ferroptosis-related genes (FRGs) was performed in 1322 patients with BCa from four independent patient cohorts and a pan-cancer cohort of 9824 patients. Twelve FRGs were selected through machine learning algorithm to construct the prognosis model. Significantly differential survival outcomes (hazard ratio (HR) = 2.09, 95% confidence interval (CI): 1.55–2.82, *p* < 0.0001) were observed between patients with high and low ferroptosis scores in the TCGA cohort, which was also verified in the E-MTAB-4321 cohort (HR = 4.71, 95% CI: 1.58–14.03, *p* < 0.0001), the GSE31684 cohort (HR = 1.76, 95% CI: 1.08–2.87, *p* = 0.02), and the pan-cancer cohort (HR = 1.15, 95% CI: 1.07–1.24, *p* < 0.0001). Tumor immunity-related pathways, including the IL-17 signaling pathway and JAK-STAT signaling pathway, were found to be associated with the ferroptosis score in BCa through a functional enrichment analysis. Further verification in the IMvigor210 cohort revealed the BCa patients with high ferroptosis scores tended to have worse survival outcome after receiving tumor immunotherapy. Significantly different ferroptosis scores could also be found between BCa patients with different reactions to treatment with immune checkpoint inhibitors.

## 1. Introduction

As one of the most aggressive malignancies worldwide, bladder cancer (BCa) was reported to have 83,730 new cases and 17,200 related deaths in the United States in 2021 [1]; in China, 80,500 new cancer cases and 32,900 related deaths in bladder were estimated in 2015 [2]. In addition, about 12.5% of patients primarily diagnosed as non-muscle invasive BCa could still suffer from disease progression after initial treatment [3,4], which greatly increases the mortality rate. For now, it is still important to identify effective prognostic markers for patients with BCa.

Currently, ferroptosis is one of the newly reported types of cell death modality that might act as an important regulator in cancer biology [5]. Being distinct from other types of cell death, ferroptosis is mainly caused by the lethal accumulation of iron-dependent lipid hydroperoxides [6]. Even though the process by which ferroptosis interacts with other types of cell death in tumorigenesis is not fully understood [7], it is well accepted that the execution of ferroptosis requires the oxidation of polyunsaturated fatty acids [5]. Moreover, ferroptosis might take part in tumor suppression dominated by *TP53* [8]. However, the roles FRGs play in BCa are still poorly understood.

In this study, we firstly explored the important roles of FRGs in BCa, and then developed and verified a novel prognostic model based on FRGs in three independent patient cohorts by using a machine learning method. Subsequently, we performed functional enrichment analysis to explore potential mechanisms associated with the FRGs’ signatures and the potential use of the ferroptosis-related signatures for predicting immune responses in patients with BCa.

## 2. Materials and Methods

### 2.1. Patient Cohorts

Four independent patient cohorts from The Cancer Genome Atlas (TCGA), ArrayExpress (E-MTAB-4321) [9], Gene Expression Omnibus (GSE31684) [10], and IMvigor 210 trial [11] were recruited in this study. Only patients with complete gene expression data and clinical prognosis information were selected for analysis. The TCGA cohort included 405 BCa samples and 19 paired normal bladder samples from radical cystectomy with pre-processed RNA-sequencing data and corresponding clinical data, which were retrieved from TCGA database. In the GSE31684 cohort, raw CEL microarray data of 93 patients managed by radical cystectomy were analyzed for background adjustment and normalization based on the Affymetrix Human Genome U133 Plus 2.0 Array through robust multichip average methods [12]. Records for 476 patients managed by radical cystectomy or transurethral resection in the E-MTAB-4321 cohort with complete clinical data and processed RNA-seq sequencing data were also downloaded from ArrayExpress. Clinicopathological and processed gene expression data of 348 mUC patients treated with immune checkpoint inhibitors (ICIs) in the IMvigor210 cohort was retrieved from IMvigor210CoreBiologies, a free data resource based on the R environment [13]. Basic clinicopathologic features are shown in Table S1 in the Supplementary Materials. In addition, 19 ferroptosis-related genes (FRGs) were retrieved from previous published studies [14]. The pan-cancer data of 32 types of malignancies, including normalized RNA-seq data and survival information for 9824 patients, were also downloaded from the TCGA database.

### 2.2. Construction and Evaluation of the Prognosis Model Based on FRGs

To determine the specific FRGs for the construction of the prognosis model for patients with BCa, we carried out a least absolute shrinkage and selection operator analysis via the glmnet package in the R environment using the training cohort (TCGA cohort), with a lambda number of 1000 to ensure the robustness of the model. Only specific FRGs associated with clinical prognosis were screened out, and their respective coefficients were also calculated. Then, we calculated the ferroptosis score as follows:

$$\mathrm{Ferroptosis}\;\mathrm{score}\;=\sum_{i=1}^{n}\left(\mathrm{Coefficient}\;\times \;\mathrm{Feri}\right)$$

The prognosis model was further verified in the independent E-MTAB-4321 cohort, GSE31684 cohort, and pan-cancer cohort with a cut-off value of the median value for each cohort.

### 2.3. Gene Set Enrichment Analysis

In order to determine the potential mechanisms related to the ferroptosis score, we carried out a gene set enrichment analysis (GSEA) [15,16] via the clusterProfiler package [17] in R; this analysis was performed based on the Kyoto Encyclopedia of Genes and Genomes (KEGG) PATHWAY database and a gene ontology (GO) analysis. The abundances of 22 types of immune cells were estimated from transcriptomic data by using CIBERSORT [18].

### 2.4. Tumor Immune Microenvironment Analysis

We estimated the abundances of various immune cells for each sample using CIBERSORT [19]. The expressions of chemokines, interleukins, interferons, MHC molecules, costimulators, coinhibitors, and other important cytokines were analyzed between patients with high and low ferroptosis scores. Key immune characteristics, including TGF-β response, IFN-γ response, macrophage regulation, proliferation, wound healing, Th1 cells, Th2 cells, and Th17 cells, were also retrieved from previous research for comparative analyses [20]. The association of the machine-learning-based ferroptosis score and the response to immunotherapy was also explored in the IMvigor210 cohort.

### 2.5. Exploring the Potential Compounds Targeting the FRG Signature

Exploration of the potential compounds that targeted the ferroptosis-related gene signature was carried out based on a dataset downloaded from CellMiner [21], which is a database and query tool designed for the cancer research community to facilitate integration and study of molecular and pharmacological data for the NCI-60 cancerous cell lines. The half-maximal inhibitory concentration (IC50) was used to predict the drug sensitivity of potential compounds.

### 2.6. Statistical Analysis

Statistical analysis was performed in R (3.6.2) for this study. Survival analysis was carried out through Kaplan–Meier curve and Cox regression analyses to compare overall survival (OS, deaths from all causes) and disease-free survival (DFS, survival without BCa-related events) with a hazard ratio (HR) and 95% confidence interval (CI). The predictive nomogram was constructed using the nomogramEx and rms packages in R, and evaluated via using concordance index (C-index) and receiver operating characteristic (ROC) curves with an area under curve (AUC) value.

## 3. Results

### 3.1. Clinicopathological Characteristics of Patients in this Study

A total of 1322 patients with BCa were included in this study. The clinicopathological features of patients in the TCGA cohort (*n* = 405), the E-MTAB-4321 cohort (*n* = 476), the GSE31684 cohort (*n* = 93), and the IMvigor210 cohort (*n* = 348) are shown in Table S1 in the Supplementary Materials, with mean follow-up duration of 27.0 ± 27.9 months, 33.1 ± 17.3 months, 47.5 ± 44.8 months and 10.2 ± 7.7 months, respectively. In the IMvigor210 cohort, a total of 348 BCa patients with follow-up information were treated with ICIs, in which 298 patients had exact immunotherapy results: complete response (CR), partial response (PR), stable disease (SD), or progressive disease (PD).

### 3.2. Construction and Verification of the Prognosis Model in Multiple Patient Cohorts

**Figure 1:**
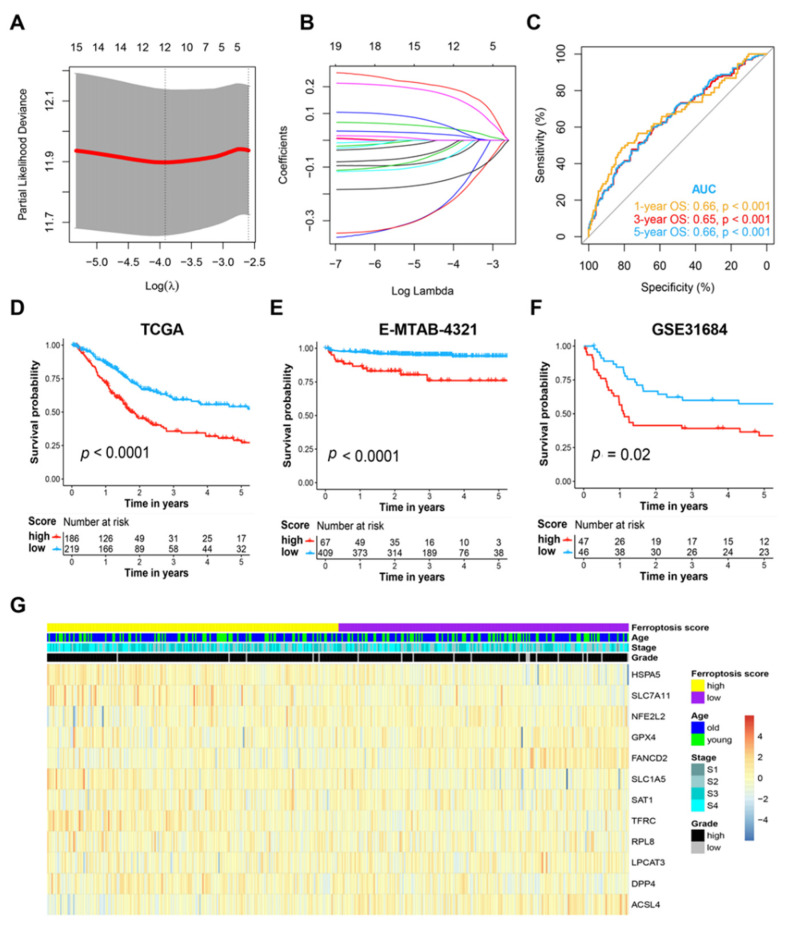
Prognosis model based on ferroptosis-related genes for BCa. (**A**,**B**) The 10-fold cross-validated error and coefficients at varying levels of penalization plotted against the log (lambda) sequence for the least absolute shrinkage and selection operator analysis, respectively. (**C**) ROC curve of 1-, 3-, and 5-year OS prediction based on the ferroptosis score. (**D**) Kaplan-Meier survival analysis of OS stratified by ferroptosis score for BCa patients in the TCGA cohort. (**E**) Kaplan-Meier survival analysis of DFS stratified by ferroptosis score in the validation E-MTAB-4321 cohort. (**F**) Kaplan-Meier survival analysis of OS stratified by ferroptosis score in another validation of the GSE31684 cohort. (**G**) Heatmap illustrating the expression of the selected genes and the distribution of clinicopathologic factors in the TCGA cohort. BCa, bladder cancer; TCGA, The Cancer Genome Atlas; ROC, receiver operating characteristic; AUC, area under the curve; OS, overall survival; DFS, disease-free survival; S1, Stage I; S2, Stage II; S3, Stage III; S4, Stage IV.

By performing a least absolute shrinkage and selection operator analysis in the training cohort (TCGA cohort), we screened out 12 FRGs, including *HSPA5*, *TFRC*, *SLC7A11*, *SLC1A5*, *DPP4*, *RPL8*, *LPCAT3*, *SAT1*, *GPX4*, *ACSL4*, *NFE2L2*, and *FANCD2*, to construct the prognosis model (Figure 1A,B). The selected genes and their respective coefficients are shown in Table S2 in the Supplementary Materials.

Based on the selected FRGs and their respective weight coefficients, the ferroptosis score was then calculated according to the formula mentioned above for each patient. When the cut-off value of the ferroptosis score was set as −1.557, the model achieved the best prediction performance in prediction of the 5-year survival status through the ROC curve analysis (Figure 1C). To evaluate the prognosis model, patients in the TCGA cohort and the E-MTAB-4321 cohort were grouped into high- or low-risk groups based on the cut-off value of −1.557. The cut-off value in the GSE31684 cohort was set as the median value because most of the ferroptosis scores in this cohort were higher than −1.557, which might have been due to the worse prognosis in this cohort. We performed a Kaplan–Meier curve survival analysis and observed a significantly differential OS (HR = 2.09, 95% CI: 1.55-2.82, *p* < 0.0001) between patients with high and low ferroptosis scores in the TCGA cohort (Figure 1D). We further verified the prognosis model in other independent patient cohorts; the results indicated that the ferroptosis score was able to distinguish patients associated with a worse DFS or OS in both of the two independent cohorts, with an HR of 4.71 (95% CI: 1.58–14.03, *p* < 0.0001) in the E-MTAB-4321 cohort (Figure 1E) and 1.76 (95% CI: 1.08–2.87, *p* = 0.02) in the GSE31684 cohort (Figure 1F). Heatmap analyses revealed that higher ferroptosis scores were associated with higher BCa tumor stages (Figure 1G).

### 3.3. Evaluation of the Ferroptosis-Related Prognosis Model

**Figure 2:**
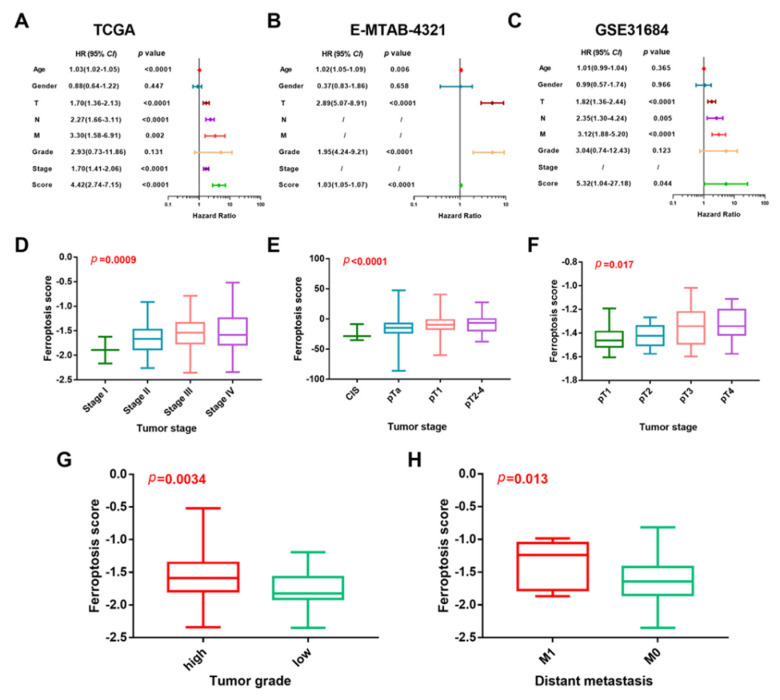
Evaluation of the ferroptosis-related prognosis model. (**A**–**C**) Univariate cox regression analyses of ferroptosis score and clinicopathologic factors in the TCGA cohort, the validation MTAB-4321 cohort, and GSE31684 cohort, respectively. (**D**–**F**) The different distributions of ferroptosis scores among different stages and lymph node metastasis status in the TCGA cohort, the validation MTAB-4321 cohort, and GSE31684 cohort, respectively. (**G**,**H**) The different distributions of ferroptosis scores between different tumor grades and distant metastasis status in the TCGA cohort. TCGA, The Cancer Genome Atlas.

**Figure 3:**
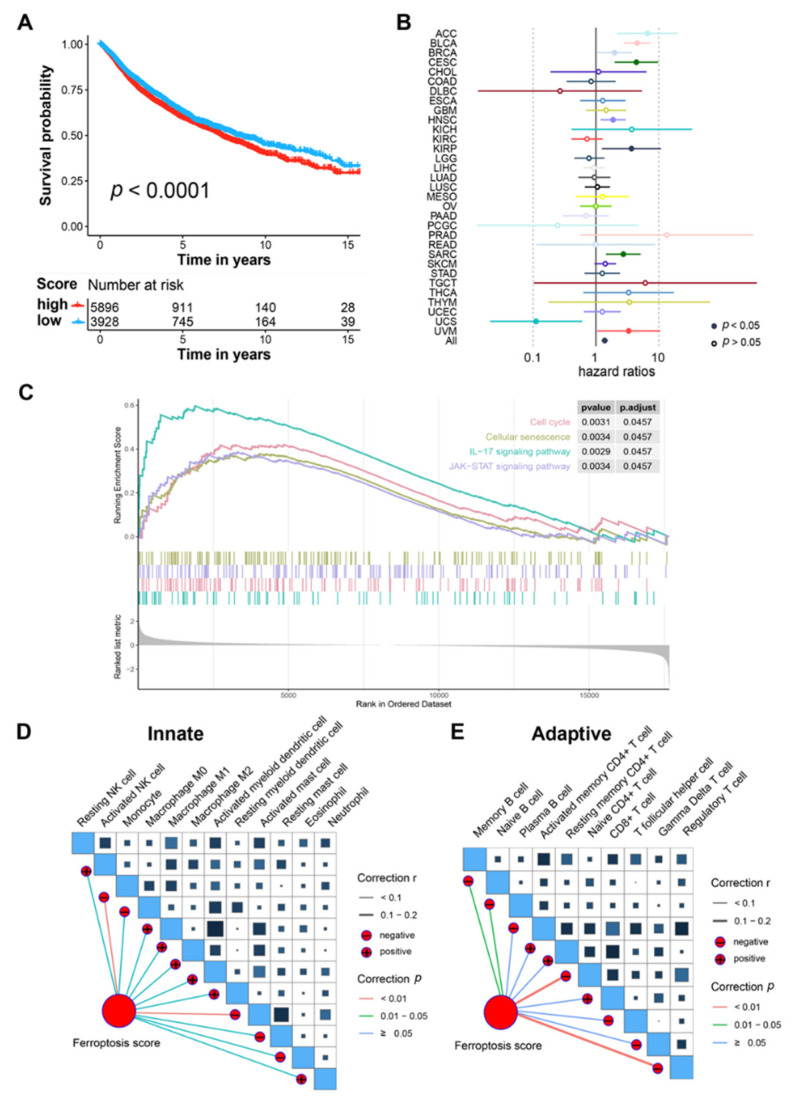
Enrichment and immunity analysis of the prognosis model in BCa. (**A**) Kaplan–Meier survival analysis of overall survival stratified by ferroptosis score for pan-cancer patients from the TCGA dataset. (**B**) Cox regression analysis of machine-learning-based ferroptosis score in different types of malignancies in the TCGA dataset. (**C**) Gene set enrichment analysis of patients with high ferroptosis scores. (**D**,**E**) Correlation analyses of ferroptosis scores and different adaptive immune cells (**D**)/innate immune cells (**E**) in the TCGA cohort. BCa, bladder cancer; TCGA, The Cancer Genome Atlas.

To further evaluate the ferroptosis-related prognosis model for patients with BCa, we performed a Cox regression analysis in three independent patient cohorts. As shown in Figure 2A–C, the ferroptosis score proved to be an independent prognostic factor for BCa patients, with an HR of 4.42, 1.03, and 5.32, respectively. Significant different distributions of ferroptosis scores could also be found among patients with different tumor stages in the TCGA cohort (Figure 2D), the E-MTAB-4321 cohort (Figure 3E), and the GSE31684 cohort (Figure 2F). In addition, a higher ferroptosis score was also found in patients with a higher tumor grade (Figure 2G) and patients with distant metastasis (Figure 2H).

### 3.4. Applying the Prognosis Model for Pan-Cancer

Next, we explored whether our prognosis model could be applied in the pan-cancer cohort. Based on 32 types of different malignancies from the TCGA database (9824 patients), our model could also significantly distinguish patients with different survival risks based on the same cut-off value of −1.557 (HR = 1.15, 95% CI: 1.07–1.24, *p* < 0.0001; Figure 3A). The Cox regression analysis illustrated that the ferroptosis score could act as an independent risk factor for multiple types of tumors, including adrenocortical carcinoma (ACC), BCa, breast invasive carcinoma (BRCA), cervical squamous cell carcinoma and endocervical adenocarcinoma (CESC), head and neck squamous cell carcinoma (NHSC), kidney renal papillary cell carcinoma (KIRP), sarcoma (SARC), and uveal melanoma (UVM), indicating the robust prediction performance of the machine-learning-based prognosis model (Figure 3B).

### 3.5. Tumor Immunity-Related Pathways Were Associated with the Ferroptosis Score in BCa

**Figure 4:**
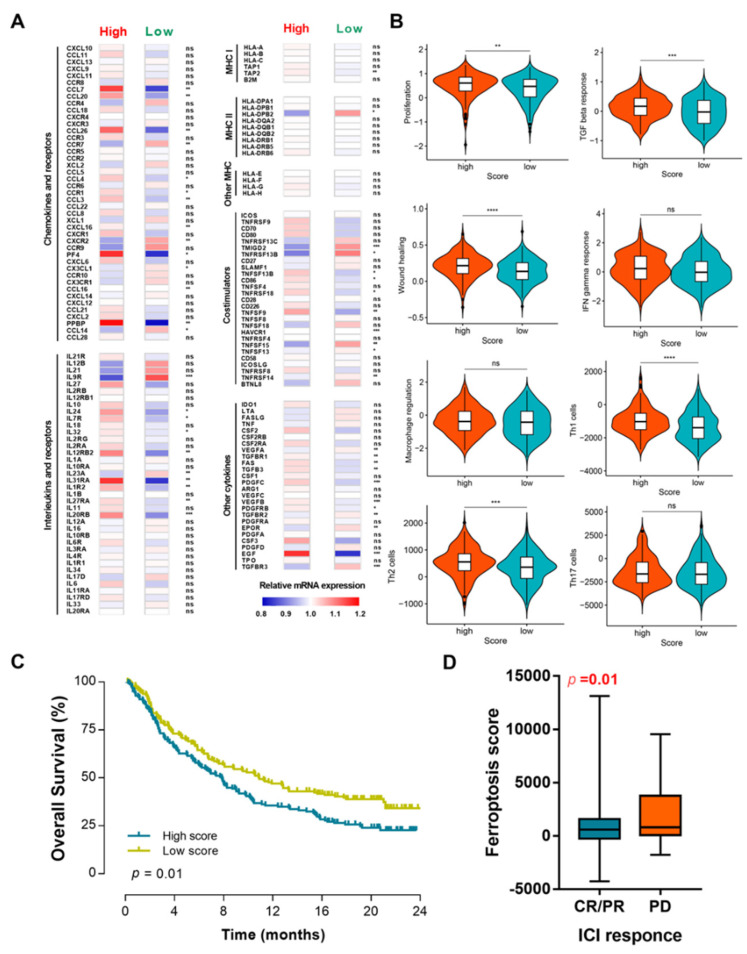
Functional enrichment analysis of ferroptosis score. (**A**) Expression of chemokines, interleukins, interferons, MHC molecules, costimulators, coinhibitors, and other important cytokines and their receptors in ccRCC from different subclusters. (**B**) Different values for vital immune characteristics in different subclusters. (**C**) Kaplan–Meier survival analysis of OS stratified by ferroptosis score in the IMvigor210 cohort treated with ICI. (**D**) Comparison of ferroptosis scores between BCa patients with different reactions to ICI treatment. OS, overall survival; BCa, bladder cancer; ICI, immune checkpoint inhibitor; CR, complete response; PR, partial response; PD, progressive disease. *, *p* < 0.05; **, *p* < 0.01; ***, *p* < 0.001; ****, *p* < 0.0001; ns, not significant.

The GSEA revealed that several pathways, including those for IL-17 signaling, JAK-STAT signaling, the cell cycle, and cellular senescence, were significantly associated with the ferroptosis score (Figure 3C). Since IL-17 has been reported to play an important role in tumor immunity [22,23], we next explored whether our ferroptosis score was associated with tumor immunity in BCa. A correlation analysis indicated that several types of immune cells were significantly associated with the ferroptosis score (Figure 3D,E), in which regulatory T cells seemed to have the highest negative correlation. Immune-related genes, including *CCL7*, *CCL20*, *CCL26*, *PF4*, *PPBP*, *IL31RA*, *IL20RB*, and *EGF* were the most highly expressed in patients with high ferroptosis scores (Figure 4A). In addition, patients with high ferroptosis scores were significantly associated with higher levels of TGF-β response, proliferation, wound healing, Th1 cells, and Th2 cells (Figure 4B). Further verification in the IMvigor210 cohort revealed the BCa patients with high ferroptosis scores tended to have a worse survival outcome after receiving tumor immunotherapy (Figure 4C). Significantly different ferroptosis scores could also be found between BCa patients with different reactions to ICI treatment (Figure 4D).

### 3.6. Integrated Nomogram Improved the Prognosis Prediction for BCa Patients

**Figure 5:**
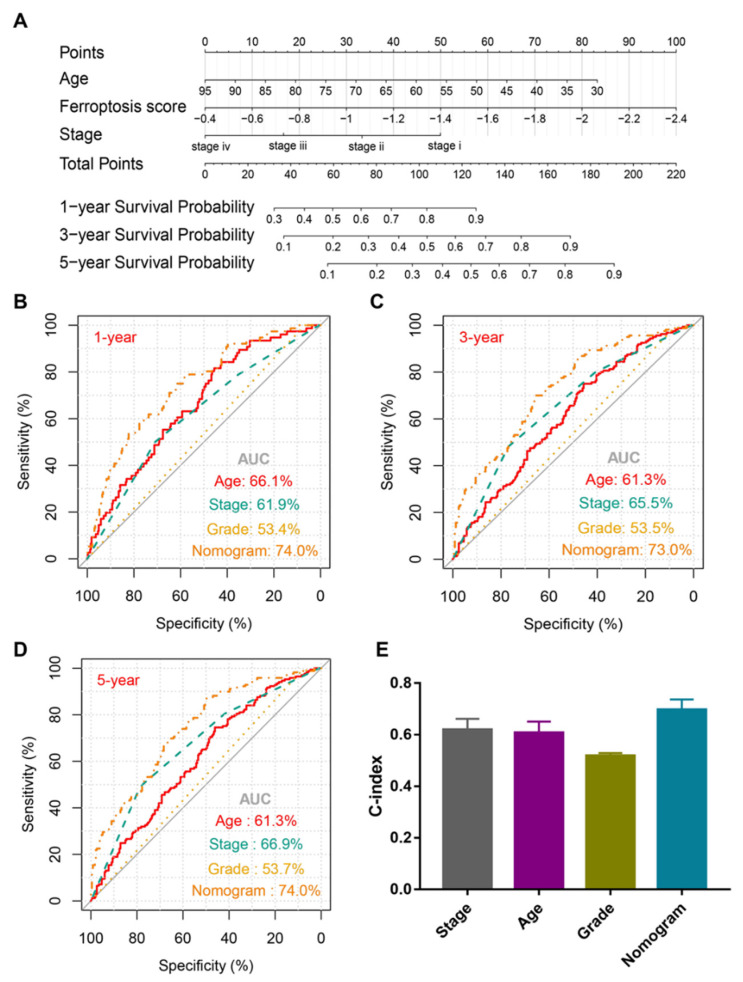
Construction and evaluation of a predictive nomogram in the TCGA cohort. (**A**) Nomogram based on ferroptosis score and clinicopathologic factors for OS prediction of BCa patients. (**B**–**D**) ROC curve of 1-, 3-, and 5-year OS prediction based on the prognostic nomogram, respectively. (**E**) Comparation of C-index among nomogram, patient age, tumor stage, and tumor grade. TCGA, The Cancer Genome Atlas; OS, overall survival; BCa, bladder cancer; ROC, receiver operating characteristic; AUC, area under curve; C-index, concordance index.

We next explored whether our ferroptosis score could be used together with current clinicopathological characteristics to improve the prognosis prediction for BCa patients. We developed an integrated nomogram based on the ferroptosis score, patient age, and tumor stage in the TCGA cohort (Figure 5A). The ROC curve analyses indicated that higher AUC values in the nomogram were found when compared with other clinicopathological characteristics (74.0% for 1-year OS, Figure 5B; 730% for 3-year OS, Figure 5C; 74.0% for 5-year OS, Figure 5D), revealing the potential clinical practicability of the nomogram.

### 3.7. Identification of Novel Candidate Compounds Targeting the Ferroptosis-Related Prognosis Model

**Figure 6:**
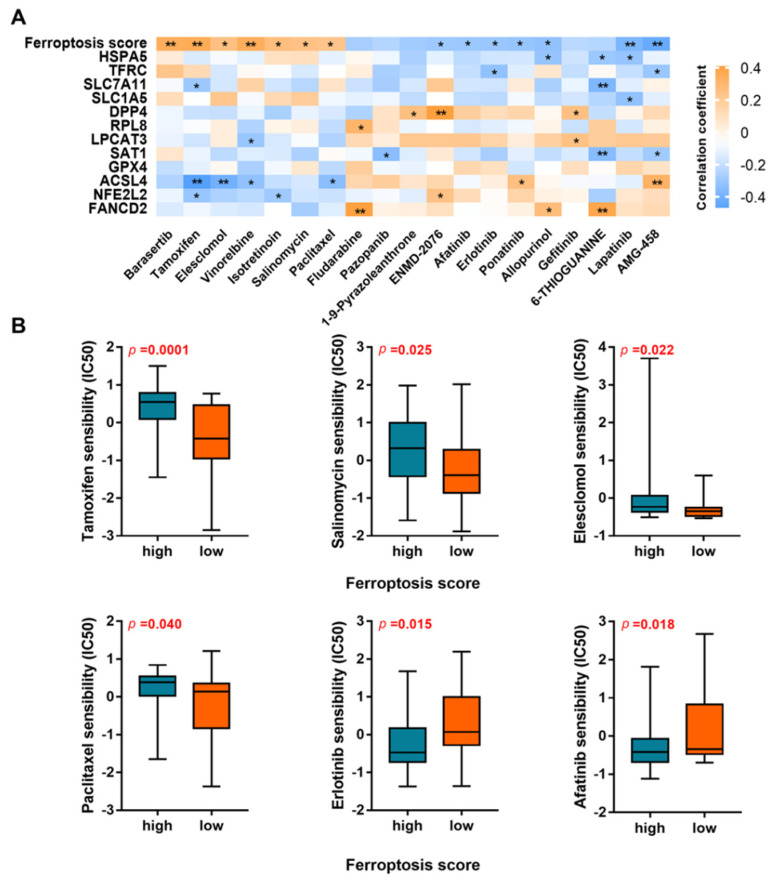
Identification of novel candidate compounds targeting the ferroptosis-related model. (**A**) Correlation analysis of compound activity of drugs and ferroptosis-related risk score/gene in NCI 60 cell line. (**B**) Comparation of drug sensibility between cell lines with high and low ferroptosis scores. IC50, half-maximal inhibitory concentration. *, *p* < 0.05; **, *p* < 0.01.

Based on the IC50 of each candidate compound, we firstly carried out a correlation analysis of the compound activity and ferroptosis-related risk score/gene in the NCI 60 cell line. As shown in Figure 6A, a total of 19 drugs were selected as candidate compounds (*p* < 0.05). Significant differences in the estimated IC50 between cancer cell lines with high and low ferroptosis scores were found in six compounds, including tamoxifen, salinomycin, elesclomol, paclitaxel, erlotinib, and afatinib (Figure 6B), which might be used as novel compounds to target the ferroptosis-related prognosis model for further analysis in patients with BCa.

## 4. Discussion

Due to the tumor heterogeneity of bladder malignancy, the accurate prediction of clinical outcome is of great importance for clinical decisions and further management for patients with BCa. As a vital physiological process that is gradually gaining attention, ferroptosis has been proved to play important roles in tumorigenesis and is significantly associated with the tumor therapeutic effect [24,25]. However, the important roles of FRGs in BCa are still poorly understood.

In this study, we constructed and verified a prognostic model based on FRGs. Effective prognosis prediction of our ferroptosis score was verified in three different patient cohorts, with HR values of 1.90, 2.63, and 1.76, respectively. Furthermore, our ferroptosis score proved to be a risk factor of OS for BCa patients. The integrated nomogram based on the ferroptosis score and clinicopathologic features performed well in predicting clinical outcome at the 1-, 3- and 5-year follow-up.

Cisplatin-based traditional chemotherapy is recommended as the first-line drug for metastatic BCa patients. [26] However, for relapsed patients, there is no approved second-line drug. Currently, with their wide application in melanoma, colorectal cancer, non-small-cell lung cancer, and Hodgkin’s lymphoma, ICI therapies targeting PD-1/PD-L1 show potential in treating relapsed BCa patients. Nevertheless, according to the results of some clinical trials, the overall response rates of PD-1/PD-L1-related immunotherapy are still improvable, and vary among individuals. [27,28] In addition, there were even some cases showing hyperprogressive disease, meaning patients showed rapid progress after PD-1/PD-L1 related immunotherapy. [29] Therefore, it is of great urgency to determine clinically useful biomarkers for predicting the clinical and ICI treatment outcomes of bladder cancer patients.

In addition to the application of immunochemistry using PD-1/PD-L1 antibodies, tumor mutational burden [30] and microsatellite instability [31] are currently two novel indicators for predicting ICI treatment. Nevertheless, both of the two biomarkers are detected through high-throughput sequencing methods, which require freshly resected samples and lead to high-level medical costs. Therefore, it is necessary to find a convenient and comparatively reliable method for predicting immunotherapy responsiveness.

In our study, our ferroptosis score was found to be significantly different between BCa patients with different reactions to ICI treatments, which could act as a potential biomarker for tumor immunotherapy of BCa. Intriguingly, the ferroptosis score in our study was correlated with abundances of some immune cells, especially regulatory T cells. Current studies reported that regulatory T cells might play important roles in the tumor environment in successful immune checkpoint therapy [32]. In addition, functional fragility of regulatory T cells is required for a response to the PD-1 blockade [33]. Therefore, the potential mechanisms underlining the ferroptosis score and regulatory T cells in immunotherapy of BCa are worth further exploration.

There were also some limitations to our study. Firstly, only public patient cohorts were included in this study, which might have resulted in a potential bias in the retrospective study. For example, some patients with BCa might receive intravesical therapies before surgery, which could stimulate an immune reaction and thus could affect gene expression at the tumor level. Secondly, the lack of clinical data regarding the population also could have caused bias in the study. Thirdly, even though high-throughput genetic techniques were tested and verified in three independent patient cohorts, experimental studies for potential mechanism exploration are still necessary for further verification of the FRG-related model.

## 5. Conclusions

In conclusion, we developed and verified a prognostic model based on FRGs, which could act as an independent risk factor for patients with BCa. The integrated nomogram based on the ferroptosis score and clinicopathologic features could improve the prognosis prediction for BCa. Tumor immunity-related pathways were also found to be associated with the ferroptosis score, and might serve as a potential biomarker for immune checkpoint therapy for BCa. However, a prospective study and the exploration of mechanisms are still needed for further analysis.

## Data Availability

The original data for this study can be found in the TCGA data portal (https://portal.gdc.cancer.gov (accessed on 12 May 2021)), ArrayExpress (https://www.ebi.ac.uk/arrayexpress (accessed on 12 May 2021)), Gene Expression Omnibus (https://www.ncbi.nlm.nih.gov/geo (accessed on 12 May 2021)), and IMvigor210CoreBiologies (http://research-pub.gene.com/IMvigor210CoreBiologies (accessed on 12 May 2021)).

## Supplementary Materials

The following supporting information can be downloaded at: https://www.mdpi.com/article/10.3390/genes13061073/s1, Table S1: Basic clinical characteristics of patients in this study; Table S2: Selected features and associated weights in the prognosis model from the TCGA cohort.
