# Supplementary material for: Robust Prediction of Prognosis and Immunotherapy Response for Bladder Cancer through Machine Learning Algorithm
Source: Genes (Basel). 2022 Jun 16;13(6):1073. doi: 10.3390/genes13061073 (PMC9223035; doi:10.3390/genes13061073)
Supplement: Supplementary file 1 [file genes-13-01073-s001.zip › genes-1713467-supplementary.pdf]

Table S1. Basic clinical characteristics of patients in this study.

	TCGA (405)	E-MTAB-4321 (476)	GSE31684 (93)	IMvigor210 (348)
**Age(years)**				
⩾65	254(62.7%)	309(64.9%)	65(69.6%)	/
< 65	150(37.0%)	167(35.1%)	28(30.4%)	/
Unknown	1(0.3%)	/	/	348(100%)
**Sex**				
Male	299(73.8%)	367(77.1%)	68(73.1%)	272(78.2%)
Female	106(26.2%)	109(22.9%)	25(26.9%)	76(21.8%)
**Grade**				
High	382(94.3%)	192(40.3%)	87(93.5%)	/
Low	20(4.9%)	277(58.2%)	6(6.5%)	/
Unknown	3(0.8%)	/	/	348(100%)
**Stage**				
I	2(0.5%)	/	/	/
II	129(31.9%)	/	/	/
III	139(34.3%)	/	/	/
IV	133(32.8%)	/	/	/
Unknown	2(0.5%)	/	/	348(100%)
**T stage**				
T1	4(1.0%)	112(23.5%)	15(16.1%)	/
T2	118(29.1%)	/	17(18.3%)	/
T3	192(47.4%)	/	42(45.2%)	/
T4	58(14.3%)	/	19(20.4%)	/
CIS	/	3(0.6%)	/	/
Ta	/	345(72.5%)	/	/
T2-4	/	16(3.4%)	/	/
Unknown	33(8.1%)	/	/	348(100%)
**N stage**				
N1	128(31.6%)	/	28(30.1%)	60(17.2%)
N0	235(58.0%)	/	49(52.7%)	288(82.8%)
Unknown	42(10.4%)	/	16(17.2%)	/
**M stage**				
M1	11(2.7%)	/	36(38.7%)	256(73.6%)
M0	195(48.1%)	/	57(61.3%)	92(26.4%)
Unknown	199(49.1%)	/	/	/
**OS status**				
Dead	178(44.0%)	/	65(69.9%)	232(66.7%)
Living	227(56.0%)	/	28(30.1%)	116(33.3%)
**DFS status**				
With cancer	/	31(6.5%)	/	/
Without cancer	/	445(93.5%)	/	/

CIS, carcinoma in situ; OS, Overall survival; DFS, Disease-free survival.

Table S2. Selected features and associated weights in the prognosis model from the TCGA cohort.

**Genes**	**Weights**
HSPA5	0.18745
TFRC	0.155169
SLC7A11	0.04036
SLC1A5	0.038926
DPP4	0.01807
RPL8	-0.00496
LPCAT3	-0.01444
SAT1	-0.04615
GPX4	-0.05375
ACSL4	-0.13698
NFE2L2	-0.19381
FANCD2	-0.21111
